# Laparoscopic Versus Open Surgical Repair of Anorectal Malformations: A Systematic Review and Meta-Analysis

**DOI:** 10.1177/30502225251401658

**Published:** 2025-12-12

**Authors:** Amani N. Alansari, Marwa Messaoud, Hanan Youssif Mohamed, Mohamed Sayed Zaazouee, Salma Mani, Ksia Amine

**Affiliations:** 1 Hamad Medical Corporation, Doha, Qatar; 2 Fattouma Bourguiba University Hospital, Monastir, Tunisia; 3 University of Monastir, Tunisia; 4 Benghazi Medical Center, Libya; 5 Al-Azhar University, Assiut, Egypt

**Keywords:** anorectal malformations, ARMs, laparoscopic, LAARP, PSARP, meta-analysis

## Abstract

**Background:**

Laparoscopic-assisted anorectoplasty (LAARP) and posterior sagittal anorectoplasty (PSARP) are established procedures for the treatment of high and intermediate anorectal malformations (ARMs). Their comparative outcomes remain under investigation.

**Objective:**

To compare the outcomes of LAARP and PSARP in children with high and intermediate ARMs.

**Methods:**

PubMed, Web of Science, Scopus, and the Cochrane Library were searched from database inception up to April 2025 for prospective studies comparing LAARP and PSARP. Data were analyzed using Review Manager (RevMan) 5.4. A fixed-effect model was primarily used for data synthesis. However, a random-effects model was applied in cases of significant heterogeneity.

**Results:**

Eight studies were included, of which 6 contributed data to the quantitative synthesis. LAARP demonstrated significantly better functional continence scores than PSARP at 1-year follow-up (SMD = 0.58; 95% CI: 0.08-1.08; *P* = .02; *I*^2^ = 34%). Operative time was longer in the LAARP group, though not statistically significant (MD = 133.16 minutes; 95% CI: −26.45 to 292.77; *P* = .10; *I*^2^ = 96%). The length of hospital stay was significantly shorter in the LAARP group (MD = −3.45 days; 95% CI: −4.47 to −2.61; *P* < .00001; *I*^2^ = 54%). LAARP was associated with potentially higher rates of anal stenosis (RR = 1.48; 95% CI: 0.62-3.55; *P* = .38; *I*^2^ = 4%) and rectal prolapse (RR = 2.15; 95% CI: 0.84-5.48; *P* = .11; *I*^2^ = 9%), though differences did not reach statistical significance.

**Conclusion:**

LAARP offers improved short-term continence and shorter hospital stay compared with PSARP in children with high and intermediate ARMs. Further large multicenter randomized trials with long-term follow-up are needed to confirm these findings.

## Introduction

Anorectal malformations (ARMs) represent a spectrum of congenital anomalies involving abnormal development of the distal rectum and anus, affecting one out of every 4000 to 5000 live births.^1^ These malformations have lifelong implications for continence, quality of life, and social functioning. ARMs are broadly categorized based on the position of the rectal pouch relative to the pelvic musculature. Early classifications, such as the Wingspread system, stratified ARMs into low, intermediate, and high types based on the position of the terminal rectum relative to the levator ani muscle.^2^ The Krickenbeck classification has become the preferred system, as it standardizes terminology, emphasizes the type and presence of fistulas, and aligns anatomical description with functional outcomes and surgical decision-making.^3^ The surgical management of ARMs depends on the complexity and anatomical type. Low anomalies mostly have a one-stage procedure; for example, the perineal fistula is managed by perineal anoplasty.^4,5^ Intermediate and high-type ARMs are more complex and may necessitate an abdominal approach, with a protective colostomy often performed as a preliminary step before definitive repair.^6^ Posterior sagittal anorectoplasty (PSARP), first introduced by Peña in the 1980s, revolutionized ARM repair and remains the standard surgical technique for these cases.^7^ It offers access to the anorectal region through a sagittal incision, allowing perfect rectal placement within the sphincter complex.^6^ Nevertheless, this open approach has been criticized for its invasiveness and potential limitations in achieving optimal functional continence, including perineal scarring, muscle transection, and prolonged recovery.^7,8^

To overcome the drawbacks of open approaches, laparoscopic-assisted anorectoplasty (LAARP) was introduced in 2000 by Bhandary et al^9^ as a minimally invasive alternative for high/intermediate lesions. LAARP provides enhanced intra-abdominal visualization, permits meticulous dissection of the rectal pouch and associated fistula, and enables accurate positioning of the neorectum, all while minimizing the need for extensive perineal dissection.^10,11^ Long-term outcomes remain variable, and concerns have been raised regarding the technique’s ability to consistently achieve optimal continence and avoid complications like mucosal prolapse or stenosis.^12^ Importantly, outcomes are also influenced by surgeon experience, as LAARP requires advanced laparoscopic skills and familiarity with pelvic anatomy; variation in expertise across centers may partly explain the heterogeneity of reported results. The increasing adoption of laparoscopic techniques has raised questions about the comparative efficacy and safety of LAARP versus the conventional PSARP. Some retrospective and prospective studies have explored this comparison. However, variations in designs, outcome measures, and sample sizes have hindered definitive conclusions.^12-16^ This systematic review and meta-analysis synthesize the current evidence derived exclusively from prospective studies to compare LAARP and PSARP in the surgical management of high and intermediate ARMs.

## Methods

This systematic review and meta-analysis followed the recommendations provided by the PRISMA (Preferred Reporting Items for Systematic Reviews and Meta-Analyses) 2020 guidelines.^17^ The review protocol was not registered in PROSPERO or any other database.

### Search Strategy and Study Selection

We searched PubMed, Scopus, Web of Science, and the Cochrane Library from their inception until April 10, 2025. The search strategy was as follows: (“Anorectal malform*” OR “Anorectal anomal*” OR “Anorectal atresia*” OR “Anorectal stenos*” OR “Anorectal defect*” OR “Imperforate anus” OR “Anal atresia*” OR “rectal atresia*” OR “Cloacal malform*” OR “Anorectal agenesis”) AND (laparoscop* OR endoscop* OR “Minimally invasive” OR “Open surg*” OR “conventional surg*” OR “standard surg*” OR “traditional surg*” OR “classic surg*” OR anorectoplasty). Our search was further supplemented by manual screening of the reference lists of included studies and relevant gray literature. Rayyan software was used for screening. MM and HY screened all retrieved records, and studies deemed potentially eligible were subjected to full-text review for final inclusion. Disagreement between reviewers was resolved through a consensus with a third researcher, ANA.

### Eligibility Criteria

We included studies based on PICOS criteria: the population comprised pediatric patients with high or intermediate ARMs as per Krickenbeck or Wingspread classifications; the intervention was LAARP; the comparator was PSARP; the outcomes assessed included functional continence, postoperative complications, and relevant operative details; and the study design was limited to prospective comparative studies (randomized controlled trials or prospective cohort studies) published in English. Retrospective studies, case reports, non-comparative studies, and non-English publications were excluded.

### Data Extraction and Quality Assessment

ANA, MSZ, and SM independently extracted data using a pre-designed data collection form that involved essential study details, including year of publication, study location, study design, surgical techniques compared, sample sizes, demographic details (age, sex), ARM types, associated anomalies, and follow-up duration. The study’s primary outcome was functional continence, which was assessed across the included studies using several scores: Kelly’s Score, the Continence Evaluation Questionnaire (CEQ) Score, and the Functional Continence Evaluation Questionnaire (FCEQ). Secondary outcomes included operation time, postoperative complications (excluding rectal prolapse and anal stenosis), anal stenosis, rectal prolapse, anal canal resting pressure, intact recto-anal inhibitory reflex, and length of hospital stay. Regarding resting pressure and the recto-anal inhibitory reflex, Yang et al^15^ performed these assessments under sedation using an anorectal pressure monitoring system after administering 6% oral chloral hydrate. Gupta et al^16^ conducted manometry at 6 months post-colostomy closure using the Phoenix V3 system, evaluating voluntary squeeze pressures only in children older than 36 months to ensure reliable cooperation. For randomized controlled trials, the Cochrane Risk of Bias 2.0 (RoB-2) tool was used.^18^ For cohort studies, the Newcastle-Ottawa Scale (NOS) was applied.^19^ ANA and HY independently assessed the methodological quality of the included studies. Any disagreements were resolved through discussion and consensus, with KA consulted when necessary.

### Statistical Analysis

Quantitative analysis was conducted using Review Manager (RevMan) version 5.4. Dichotomous outcomes were analyzed using risk ratios (RR), while continuous outcomes were assessed using mean differences (MD) or standardized mean differences (SMD), each reported with 95% confidence intervals. The SMD was specifically employed to evaluate the primary outcome of functional continence, given the variability in assessment scores across studies. Subgroup analyses were conducted for this outcome based on the follow-up time points. A fixed-effect model was primarily used for data synthesis. However, a random-effects model was applied in cases of significant heterogeneity (*I*^2^ > 50%). Heterogeneity was evaluated using the I^2^ statistic, with thresholds interpreted as outlined in the Cochrane Handbook.^20^

## Results

### Search Results

**Figure 1.**
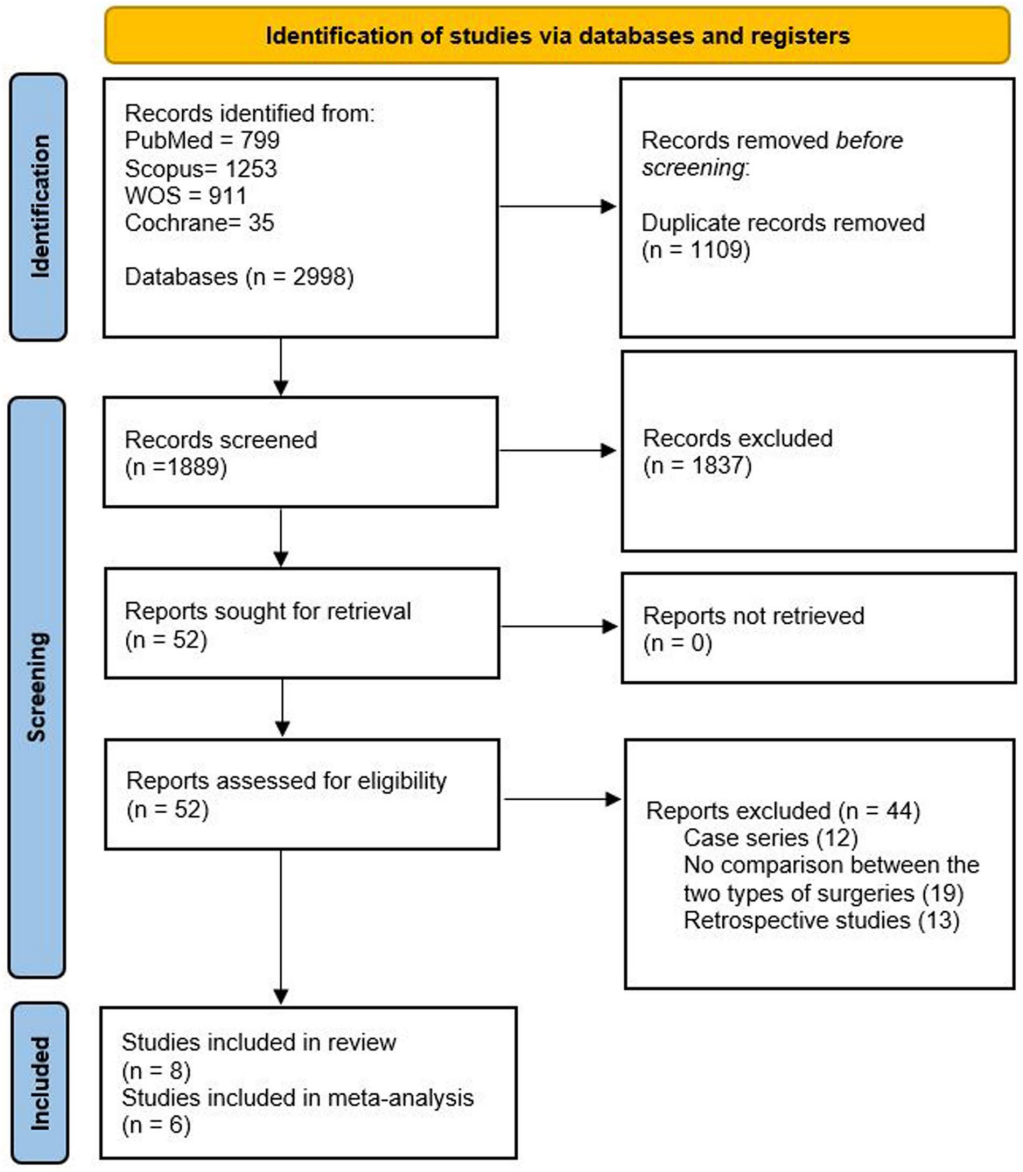
PRISMA flow chart showing the study selection process and the included studies.

Our search identified 2998 records. After removing duplicates, 1889 studies remained for the title and abstract screening. Of these, 52 studies were identified as potentially relevant and subjected to full-text review. Ultimately, only 8 studies met the predefined inclusion criteria. Among them, 6 studies were eligible for inclusion in the meta-analysis.^14-16,21-23^ One study was excluded due to insufficient data on the commonly reported outcomes,^24^ and another was excluded because of potential overlap in the recruitment period with data from the same institution.^25^ Both studies were therefore considered only in qualitative synthesis. The PRISMA flow diagram is shown in Figure 1.

### Study Characteristics

This systematic review included 8 prospective studies with 205 children diagnosed with ARMs. The studies were conducted between 2003 and 2022 across India, Japan, China, and South Africa, comprising 2 randomized controlled trials and 6 prospective cohort studies. Sample sizes ranged from 2 to 24 patients per group, with several studies being very small and therefore at risk of being underpowered to detect true differences between techniques. The included studies compared the outcomes of LAARP to conventional open surgery approaches, primarily the PSARP. A summary of study arms and surgical techniques is demonstrated in Table 1. Study sample sizes ranged from 2 to 24 patients per group, with follow-up durations ranging from 6 months to over 7 years. The patients’ mean age at surgery ranged from neonatal (a few days old) to approximately 28 months, and most cohorts had a male predominance. Variability in the types of ARMs was observed across patient populations, including recto-bladder neck, recto-prostatic, recto-bulbar, and rectovaginal fistulae, as well as more complex cases such as cloacal anomalies and anorectal agenesis without fistula. Associated congenital anomalies were common, particularly renal, cardiac, spinal, and musculoskeletal malformations, further highlighting the complexity of this patient population. These details are summarized in Table 2 and Table S1.

**Table 1.** Summary of the Included Studies.

ID	Country	Study design	Laparoscopic surgery approach details	Open surgery approach details	Main results
Gupta 2022^16^	India	Randomized Controlled Trial	Minimally invasive anorectoplasty	Conventional posterior sagittal anorectoplasty	Similar continence; LAARP → fewer wound complications
Koga 2014^23^	Japan	Prospective cohort study	Georgeson technique; intraop catheter + cystoscopy	de Vries–Peña technique	Similar RBF outcomes; LAARP → fewer wound infections, ↑ prolapse
Pandey 2014^25^	India	Prospective cohort study	LAARP after neonatal colostomy	Single-stage repair (±APPT)	Fistula division w/o ligation ↓ diverticula risk (needs confirmation)
England 2012^14^	South Africa	Prospective cohort study	3 mm instruments, electrostimulation tract, sutured anoplasty	Standard PSARP	Anal stenosis frequent in LAARP (poor dilatation adherence main cause)
Koga 2010^22^	Japan	Prospective cohort study	Colon pull-through (Georgeson)	Peña procedure	Comparable continence; GPT less adverse functional impact
Yang 2009^15^	China	Randomized controlled study	LAARP at 2-3 months post-colostomy (Georgeson modified)	PSARP at 2-3 months post-colostomy (Peña)	Similar clinical scores; LAARP ↑ anal canal resting pressure, ↓ hospital stay
Ichijo 2008^21^	Japan	Prospective cohort study	LAARP with intraop colonoscopy/cystoscopy in later cases	PSARP, some w/ prior abdominal surgery	Similar stress/muscle outcomes; LAARP ↑ CEQ scores
Lin 2003^24^	China	Prospective cohort study	Pull-through centered in levator sling, no division of sphincter	Muscle/sphincter division for reconstruction	Early LAARP ↑ manometry results; long-term outcomes unclear

Abbreviations: LAARP, laparoscopic-assisted anorectoplasty; PSARP, posterior sagittal anorectoplasty; RBF, recto-bulbar fistula; APPT, abdominoperineal pull-through approach; GPT, Georgeson’s laparoscopy-assisted colon pull through; FCEQ, fecal continence evaluation questionnaire; PPA, Pena’s posterior sagittal anorectoplasty; CEQ, continence evaluation questionnaire.

**Table 2.** Baseline Characteristics of the Included Studies.

ID	Group	Sample size	Mean age at surgery	Sex	Follow-up period
Gupta 2022^16^	LAARP	8	11.38 ± 8.35 months	7 males, 1 female	6 months after colostomy closure
	PSARP	8	27.75 ± 32.68 months	8 males	
Koga 2014^23^	LAARP	12	7.9 ± 3.9 months	20 males	75.8 ± 49.1 months
	PSARP	8	9.0 ± 6.0 months		107.3 ± 41.6 months
Pandey 2014^25^	LAARP	2	6 months	24 males	1 year
	PSARP	22	4.23 ± 1.24 days		
England 2012^14^	LAARP	24	2.6-15.0 months	21 male, 3 female	1-5 years
	PSARP	19	4-39 months	Not reported	1-12 years
Koga 2010^22^	LAARP	20	6.6 ± 3.2 months	15 male, 5 female	7.0 ± 3.2 years
	PSARP	13	6.4 ± 4.0 months	11 male, 2 female	7.0 ± 2.2 years
Yang 2009^15^	LAARP	11	2.7 ± 0.5 months	11 males	17.4 ± 4.9 months
	PSARP	12	2.8 ± 0.4 months	8 male, 4 female	19.3 ± 6.2 months
Ichijo 2008^21^	LAARP	15	9.6 ± 7.1 months	10 males, 5 females	60.0 ± 15.1 months
	PSARP	9	7.1 ± 4.8 month	7 males, 2 females	66.3 ± 24.0 months
Lin 2003^24^	LAARP	9	3.6 months	6 male, 3 female	12.6 months
	PSARP	13	6.7 months	10 male, 3 female	10.4 months

Abbreviations: LAARP, laparoscopic-assisted anorectoplasty; PSARP, posterior sagittal anorectoplasty.

During data extraction, we identified a possible overlap in patient recruitment periods among 3 studies originating from the same institution.^21,22,25^ Because these reports shared similar timeframes and institutional settings, inclusion of all 3 could have introduced duplicate data. To minimize this risk, when an outcome was reported by more than one of these studies, only 1 dataset was retained for quantitative analysis. For functional continence, data from Koga et al^21^ were included, as this study corresponded most closely with the follow-up durations reported in other included studies, ensuring analytical consistency. The earlier study by Ichijo et al^25^ was therefore not included in any quantitative synthesis. This study analyzed 24 infants with high or intermediate-type imperforate anus treated with either LAARP or PSARP.^25^ Using anal endosonography, pelvic magnetic resonance imaging (MRI), and a structured continence evaluation questionnaire (CEQ), the authors found no significant differences in sphincter muscle thickness or MRI symmetry scores between the 2 groups. However, functional continence scores (CEQ) were generally higher after LAARP, reaching statistical significance at 3 and 4 years of follow-up (*P* < .05). Parameters of surgical stress (fever, WBC count, and CRP levels) were comparable. Overall, the study suggested that LAARP may provide slightly better midterm functional outcomes, despite equivalent anatomic findings.

The other study that was not incorporated into the meta-analysis, conducted by Pandey et al,^24^ evaluated surgical outcomes in 24 male neonates with high anorectal malformations, specifically recto-bladder neck and recto-prostatic fistulas. Patients were managed using 3 different approaches: open abdominoperineal pull-through (12 cases), posterior sagittal anorectoplasty (PSARP, 10 cases), and laparoscopic-assisted anorectoplasty (LAARP, 2 cases), which was performed at 6 months in patients who had undergone prior colostomy. Across all groups, no postoperative urinary leaks were observed, and all patients demonstrated normal findings on voiding cystourethrograms at 1-year follow-up. Despite the limited number of laparoscopic cases, the results suggested that LAARP is a safe and feasible option for selected cases of high ARM, offering outcomes comparable to those of traditional open techniques.

### Quality Assessment

According to the ROB-2 tool, both randomized trials were judged to have a low risk of bias.^15,16^ The detailed domain-level assessments are illustrated in Figures S1 and S2. For cohort studies assessed using the NOS, 5 were rated as good quality, with scores ranging from 7 to 8 out of 9, while 1 study was rated as fair quality (6/9) due to limited information on comparability.^24^ These results are presented in Table S2.

### Meta-analysis

#### Functional Outcomes

**Figure 2.**
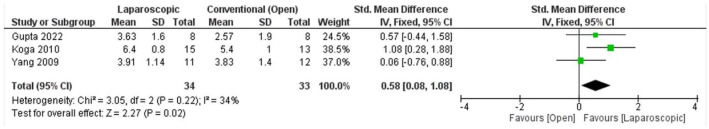
Forest plot comparing functional fecal continence scores between laparoscopic and open surgery within 1 year postoperatively.

As shown in Figure 2, the results indicate a general trend favoring laparoscopic surgery, suggesting better ***functional fecal continence scores*** than conventional open surgery. The laparoscopic approach demonstrated a statistically significant advantage in functional continence during early follow-up (within 1 year postoperatively; SMD = 0.58; 95% CI ranging from 0.08 to 1.08; *P* = .02; *I*^2^ = 34%).

Longer-term outcomes could not be quantitatively analyzed, as they were reported only in studies with overlapping cohorts. Qualitatively, these reports described a consistent pattern of improving continence scores with time and comparable anorectal anatomy between laparoscopic and open repairs, supporting the durability of functional recovery after the laparoscopic approach.^21,22,25^

The analysis did not identify any difference in ***anal canal resting pressure*** between the 2 groups (MD = 3.31; 95% CI: −0.94 to 7.56; *P* = .13; *I*^2^ = 51%; Figure S3). Similarly, no difference was found regarding the recto-anal reflex (RR = 1.37; 95% CI: 0.76-2.47; *P* = .3; *I*^2^ = 69%; Figure S4). Due to the significant heterogeneity, a leave-one-out test was applied, excluding Lin et al.’s study, which showed no appreciable difference (RR = 1.05; 95% CI: 0.77-1.43; *P* = .78; *I*^2^ = 0%; Figure S5).

#### Operative Time and Length of Hospital Stay

**Figure 3.**
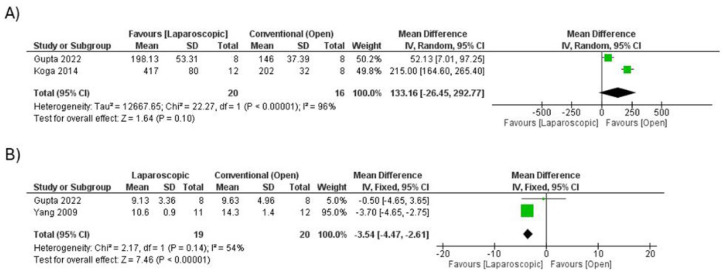
(A) Forest plot comparing the operation time between laparoscopic and open surgery. (B) Forest plot comparing the length of hospital stay between laparoscopic and open surgery.

Although ***operative time*** tended to be longer in the laparoscopic group, the difference was not statistically significant, and this outcome was derived from only 2 studies (MD = 133.16 minutes; 95% CI: −26.45 to 292.77; *P* = .1; I^2^ = 96%; Figure 3A). The extremely wide confidence interval and very high heterogeneity suggest that the effect is inconclusive and may reflect differences in surgeon expertise, operative protocols, and institutional experience with LAARP. In contrast, ***hospital length of stay***, also reported in 2 studies, was consistently shorter in the laparoscopic group (MD = −3.54 days; 95% CI: −4.47 to −2.61; *P* < .00001; *I*^2^ = 54%; Figure 3B). This finding represents stronger evidence despite moderate heterogeneity, supporting a perioperative recovery advantage with LAARP. Both outcomes should therefore be interpreted as exploratory given the small number of contributing studies.

#### Complications

**Figure 4.**
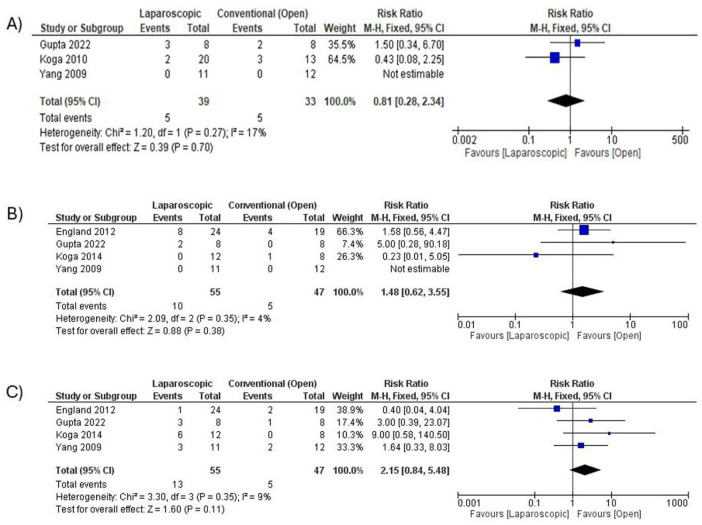
(A) Forest plot comparing overall complications (excluding anal stenosis and rectal prolapse) between laparoscopic and open surgery. (B) Forest plot comparing anal stenosis between laparoscopic and open surgery. (C) Forest plot comparing rectal prolapse between laparoscopic and open surgery.

Overall complications (***excluding anal stenosis and rectal prolapse***) showed a trend toward being lower in the laparoscopic group, although this did not reach statistical significance (RR = 0.81; 95% CI: 0.28-2.34; *P* = .70; *I*^2^ = 17%; Figure 4A). In contrast, the incidences of ***anal stenosis*** (RR = 1.48; 95% CI: 0.62-3.55; *P* = .38; *I*^2^ = 4%; Figure 4B) and ***rectal prolapse*** (RR = 2.15; 95% CI: 0.84-5.48; *P* = .11; *I*^2^ = 9%) were higher in the laparoscopic group. Although these findings were not statistically significant, the direction of effect raises clinically relevant concerns and warrants cautious interpretation in surgical decision-making (Figure 4C).

## Discussion

This systematic review and meta-analysis evaluated the outcomes of surgical management of high and intermediate ARMs with LAARP and PSARP using evidence from prospective studies exclusively. The findings show that LAARP has better short-term functional outcomes than open surgery, particularly within the first year after the procedure. Although the advantage appeared to diminish over time, early benefits in fecal continence could have meaningful implications for the quality of life and social integration of affected children. Furthermore, LAARP was associated with a shorter hospitalization. Regarding postoperative complications, anal resting pressure, and the recto-anal inhibitory reflex, both approaches demonstrated similar outcomes.

Earlier meta-analyses have attempted to compare the outcomes of LAARP versus PSARP. Han et al indicated that LAARP was associated with reduced rates of wound-related complications, higher anal canal resting pressures, a lower incidence of constipation, and a shorter length of hospital stay. However, operative time differences between the 2 techniques were inconclusive, and no significant differences were observed regarding rectal prolapse, anal stenosis, and anorectal manometry results.^26^ More recently, Miscia et al^27^ conducted a meta-analysis that demonstrated a shorter LAARP hospitalization than PSARP. Nevertheless, both were comparable in terms of early postoperative complications, incidence of rectal prolapse, voluntary bowel movements, and rates of soiling at long-term follow-up. It is important to note that both previous meta-analyses were primarily based on retrospective studies, which can introduce selection bias and inconsistencies in data reporting. In contrast, this is the first prospective-only meta-analysis, offering a more robust comparison of the 2 approaches.

The higher functional continence scores observed in the short-term follow-up after LAARP compared to PSARP can be attributed to several factors. LAARP provides superior anatomical visualization, achieving accurate placement of the rectum within the sphincter complex and significantly reducing trauma to important continence structures.^21,25^ There is also less perineal dissection achieved through the laparoscopic approach, which protects the surrounding nerves and muscles necessary for voluntary bowel control. Furthermore, because of the minimally invasive procedure, postoperative fibrosis and scarring are minimized, promoting early anorectal function. Ichijo et al observed that functional continence, assessed by the Continence Evaluation Questionnaire (CEQ) score, was generally higher in the LAARP group throughout the study. However, they did not observe differences in mean muscle thickness or magnetic resonance imaging scores.^25^ It is important to note that long-term follow-up studies often show parallel continence outcomes between LAARP and PSARP, suggesting that early advantages may lessen over time as healing and adaptation occur. Variations across different studies reflect the influence of patient anatomy, surgeon experience, and heterogeneity in assessment tools. These findings highlight that while LAARP may facilitate faster functional recovery in the early postoperative period, both techniques can ultimately achieve satisfactory long-term continence outcomes, guiding individualized surgical decision-making.

Regarding postoperative complications, the laparoscopic group demonstrated a lower overall complication rate compared to the open group, excluding anal stenosis and rectal prolapse. Reported complications in the literature include wound infection, urinary tract infections, pneumonia, and urethral diverticulum.^28^ Although rectal prolapse and anal stenosis were observed more frequently after LAARP, these differences did not reach statistical significance. The higher odds of anal stenosis were mainly attributed to 2 studies. England et al identified anal stenosis as the most common complication following LAARP, with an incidence of 33%. Poor adherence to postoperative anal dilatation protocols was the main contributor to anal stenosis, with additional roles suggested for ischemia, nutritional status, and technical factors. Despite dilatation attempts, many cases ultimately required redo-anorectoplasty.^14^ Gupta et al^16^ reported a 25% incidence of anal stenosis in the LAARP group, with 1 patient requiring redo-PSARP.

Sterne et al^18^ reported a 37% rate of rectal mucosal prolapse in the LAARP group, attributed to excessive rectal mobilization without anchoring sutures to the presacral fascia. Similarly, Pandey et al^24^ found a higher incidence of rectal mucosal prolapse after LAARP compared to PSARP (50.0% vs 0%). Interestingly, prolapse was observed only in the earlier cases, likely due to overly aggressive or extensive dissection that may have compromised blood supply and impaired tissue healing. With the refinement of the technique over time, no cases of rectal mucosal prolapse were reported in later series in this study. Thus, with meticulous surgical technique and careful rectal dissection, the risk of such complications can be significantly minimized.

This meta-analysis provides valuable insights into the comparative performance of LAARP and PSARP. It highlights the potential functional benefits of the laparoscopic approach, particularly in the early postoperative years. Nevertheless, several limitations should be acknowledged. First, the number of included studies was relatively small, and most had modest sample sizes, limiting the power of the analysis to detect differences, particularly for rare outcomes such as prolapse or stenosis. Second, although only prospective studies were included to minimize bias, heterogeneity in patient characteristics and outcome assessment tools, particularly the lack of standardized continence scoring systems across studies, could have introduced variability that may affect the generalizability of the results. Importantly, the presence of associated anomalies, especially spinal anomalies, which are known to influence functional outcomes, may have differed between groups. While this issue was considered in the included RCTs, the cohort studies may not have had matched baseline characteristics, and subgroup analysis based on associated anomalies could not be performed due to insufficient data. In addition, the included studies demonstrated geographic and temporal clustering, with most conducted in limited regions and over different time periods, which may affect the applicability of findings to broader populations. Surgeon experience and patterns of regional adoption may also have influenced outcomes, potentially introducing bias. Finally, the duration of follow-up varied substantially across studies, and data beyond 5 years postoperatively were limited, restricting the ability to draw firm conclusions regarding the long-term functional outcomes. Future research should aim for multicenter, geographically diverse trials with standardized reporting and longer follow-up to strengthen the evidence base.

## Conclusion

This meta-analysis suggests that LAARP offers superior early functional outcomes and shorter hospitalization compared to PSARP for high and intermediate anorectal malformations. LAARP was associated with a lower overall rate of postoperative complications, although rectal prolapse and anal stenosis occurred more frequently. In spite of that, these differences in complication rates did not reach statistical significance. Clinically, LAARP may be preferred as a first-line approach in centers with established laparoscopic expertise, provided that strict postoperative follow-up and adherence to anal dilatation protocols are ensured. Future research should focus on large, multicenter randomized trials with standardized outcome measures and extended follow-up of at least 10 years to clarify the durability and long-term safety of LAARP compared to PSARP.

## Supplemental Material

sj-docx-1-gph-10.1177_30502225251401658 – Supplemental material for Laparoscopic Versus Open Surgical Repair of Anorectal Malformations: A Systematic Review and Meta-Analysis Supplemental material, sj-docx-1-gph-10.1177_30502225251401658 for Laparoscopic Versus Open Surgical Repair of Anorectal Malformations: A Systematic Review and Meta-Analysis by Amani N. Alansari, Marwa Messaoud, Hanan Youssif Mohamed, Mohamed Sayed Zaazouee, Salma Mani and Ksia Amine in Sage Open Pediatrics
