# Supplementary material for: Laparoscopic Versus Open Surgical Repair of Anorectal Malformations: A Systematic Review and Meta-Analysis
Source: Sage Open Pediatr. 2025 Dec 12;12:30502225251401658. doi: 10.1177/30502225251401658 (PMC12701234; doi:10.1177/30502225251401658)
Supplement: sj-docx-1-gph-10.1177_30502225251401658 – Supplemental material for Laparoscopic Versus Open Surgical Repair of Anorectal Malformations: A Systematic Review and Meta-Analysis [file sj-docx-1-gph-10.1177_30502225251401658.docx]

**Table S1. Details of ARMs and the associated anomalies.**

| ID | Group | **Anorectal malformation type** | **Associated anomalies** |
| --- | --- | --- | --- |
| Gupta 2022 [16] | LAARP | Recto-bladder neck fistula: 4 Recto-prostatic fistula: 1 Recto-bulbar fistula: 1 Rectovaginal fistula: 1 No fistula (high pouch): 1 | Renal anomalies: 3 Spinal anomalies: 1 Cardiac anomalies: 1 Musculoskeletal anomalies: 1 GI anomalies: 0 Other anomalies: 3 |
|  | PSARP | Recto-bladder neck fistula: 4 Recto-prostatic fistula: 2 Recto-bulbar fistula: 2 Rectovaginal fistula: 0 No fistula (high pouch): 0 | Renal anomalies: 1 Spinal anomalies: 2 Cardiac anomalies: 2 Musculoskeletal anomalies: 1 GI anomalies: 1 Other anomalies: 2 |
| Koga 2014 [23] | LAARP | Male imperforate anus with recto-bulbar fistula | Sacral ratio <0.4: 1 Tethered cord: 1 |
|  | PSARP |  | Sacral ratio <0.4: 1 Tethered cord: 1 |
| Pandey 2014 [25] | LAARP | Recto-bladder neck fistula (16 cases),  Recto-prostatic fistula (8) | Mild bilateral hydroureteronephrosis: 2 |
|  | PSARP |  |  |
| England 2012 [14] | LAARP | Vestibular fistula: 2 Bulbar fistula: 9 Prostatic fistula: 7 Vesical fistula: 3 No fistula: 3 | 16/24 LAARP patients;  Renal: 7  Cardiac: 4  Vertebral: 2  Limb: 1  Other (dysmorphism, rib fusion): 2 |
|  | PSARP | Vestibular fistula: 3 Bulbar fistula: 4 Prostatic fistula: 5 Vesical fistula: 3 No fistula: 4 |  |
| Koga 2010 [22] | LAARP | Recto-prostatic urethral fistula,  Recto-bulbar urethral fistula, Anorectal agenesis without fistula, Rectovaginal fistula, Recto-vestibular fistula with absent vagina,  Cloacal anomaly,  Rectovesical fistula | Not reported |
|  | PSARP |  |  |
| Yang 2009 [15] | LAARP | Rectourethral fistula: 2 Recto-prostatic-urethral fistula: 3 Rectovesical fistula: 3 Rectovaginal fistula: 0 Anorectal agenesis: 3 | Not reported |
|  | PSARP | Rectourethral fistula: 4 Recto-prostatic-urethral fistula: 2 Rectovesical fistula: 1 Rectovaginal fistula: 3 Anorectal agenesis: 2 |  |
| Ichijo 2008 [21] | LAARP | Males with recto-prostatic urethral fistula: 5  Males with recto-bulbar urethral fistula: 4  Male with anorectal agenesis without fistula: 1  Female with rectovaginal fistula: 1  Females with recto-vestibular fistula with absent vagina: 2  Females with cloacal anomaly: 2  Males with rectovesical fistula: 2  Males with recto-bulbar urethral fistula: 4  Male with anorectal agenesis without fistula: 1  Females with recto-vestibular fistula: 2 | Malrotation, duodenal atresia (in some open surgery cases) |
|  | PSARP | Males with rectovesical fistula: 2  Males with recto-bulbar urethral fistula: 4  Male with anorectal agenesis without fistula: 1  Females with recto-vestibular fistula: 2 |  |
| Lin 2003 [24] | LAARP | Recto-prostatic fistula (5),  Recto-bulbar fistula (1),  Rectovaginal fistula (3) | Down syndrome:1  Hypospadias: 1  Congenital heart disease: 2 |
|  | PSARP | Recto-prostatic fistula: 7  Recto-bulbar fistula: 2  Rectovaginal fistula: 3  Blind pouch: 1 | Penoscrotal transposition and hypospadias: 1  Hypospadias: 1  Esophageal atresia with tracheoesophageal fistula: 1  Down syndrome: 1 |

**Table S2.** Quality assessment of cohort studies using the NOS tool.

| Study ID | Selection | | | | | Comparability | Outcome | | | | Overall |
| --- | --- | --- | --- | --- | --- | --- | --- | --- | --- | --- | --- |
|  | D1 | D2 | D3 | D4 |  |  | D5 | D6 | D7 |  |  |
| Koga 2014 | * | * | * | * |  |  | * | * | * |  | Good |
| Pandey 2014 | * |  | * | * |  |  | * | * | * |  | Fair |
| England 2012 | * | * | * | * |  | * | * | * |  |  | Good |
| Koga 2010 | * | * | * | * |  | * | * | * |  |  | Good |
| Ichijo 2008 | * | * | * | * |  | * | * | * | * |  | Good |
| Lin 2003 | * | * | * | * |  | * | * | * |  |  | Good |

D1: Is the case definition adequate/Representative of the exposed cohort?

D2: Representative of the cases/Selection of the non-exposed cohort.

D3: Selection of Controls/Ascertainment of exposure.

D4: Definition of Controls/ Demonstration that outcome of interest was not present at start of study.

D5: Ascertainment of exposure/ Assessment of outcome.

D6: Same method of ascertainment for cases and controls/ Was follow-up long enough for outcomes to occur.

D7: Non-Response rate/ Adequacy of follow up of cohorts.


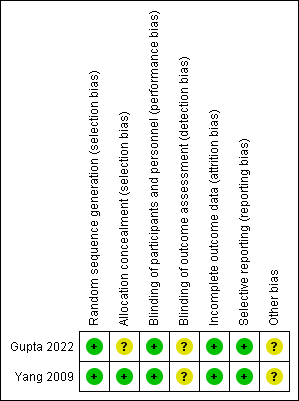


**Figure S1:** Risk of bias assessment for individual randomized controlled trials (RoB 2.0 domains shown with color coding: green = low risk, yellow = unclear risk, red = high risk).


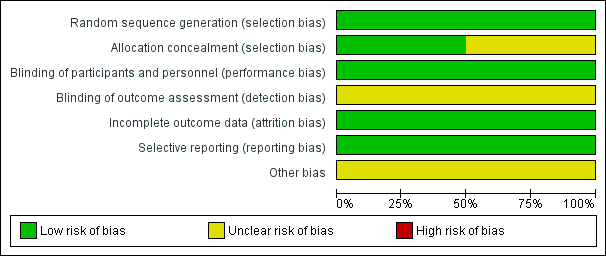


**Figure S2**: Summary risk of bias assessment across included randomized controlled trials (proportion of studies rated as low, unclear, or high risk for each domain).


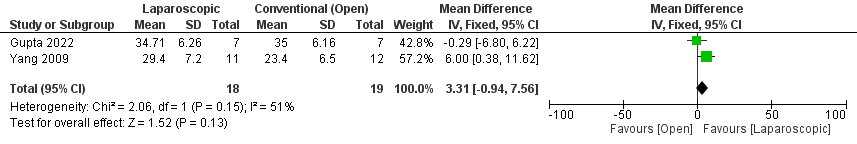


**Figure S3.** Forest plot comparing post-surgery resting anal pressure between laparoscopic and open surgery.


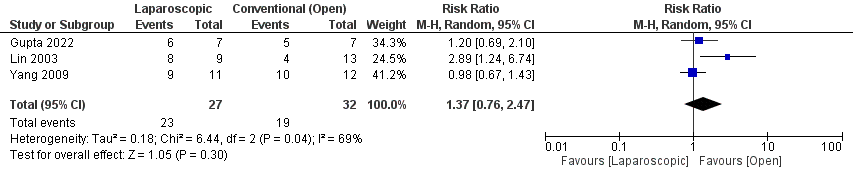


**Figure S4.** Forest plot comparing post-surgery recto0-anal reflex between laparoscopic and open surgery. (Before sensitivity)


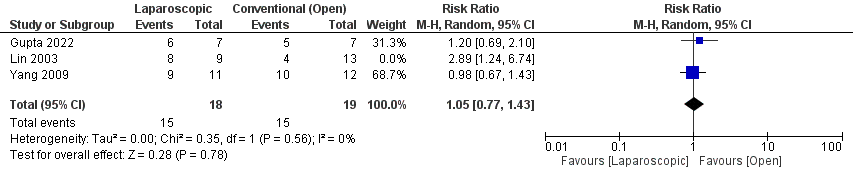


**Figure S5.** Forest plot comparing post-surgery recto0-anal reflex between laparoscopic and open surgery. (After sensitivity)
